# Microbial Conversion of Shrimp Heads to Proteases and Chitin as an Effective Dye Adsorbent

**DOI:** 10.3390/polym12102228

**Published:** 2020-09-28

**Authors:** Chien Thang Doan, Thi Ngoc Tran, Chuan-Lu Wang, San-Lang Wang

**Affiliations:** 1 Department of Natural Science and Technology, Tay Nguyen University, Buon Ma Thuot 630000, Vietnam; dcthang@ttn.edu.vn (C.T.D.); ttngoc@ttn.edu.vn (T.N.T.); 2 Department of Chemistry, Tamkang University, New Taipei City 25137, Taiwan; 3 Department of Fashion Beauty Design, Lan Yang Institute of Technology, Yilan County 26141, Taiwan; chuanlu@mail.fit.edu.tw; 4 Life Science Development Center, Tamkang University, New Taipei City 25137, Taiwan

**Keywords:** shrimp heads, chitin, dye adsorbent, protease, *Paenibacillus*

## Abstract

As a green and effective technique in the production of a large number of valuable products, the microbial conversion of chitinous fishery wastes is receiving much attention. In this study, protease production using the *Paenibacillus mucilaginosus* TKU032 strain was conducted on culture media containing several common types of chitinous fishery by-products serving as the carbon and nitrogen (C/N) nutrition source. Among the chitinous wastes, 1.5% (w/v) shrimp head powder (SHP) was found to be the most appropriate nutritional source for protease production when a maximal enzyme activity of 3.14 ± 0.1 U/mL was observed on the 3rd day of the culture period. The molecular mass of *P. mucilaginosus* TKU032 protease was estimated to be nearly 32 kDa by the polyacrylamide gel electrophoresis method. The residual SHP obtained from the culture medium was also considered to be utilized for chitin extraction. The deproteinization rate of the fermentation was estimated to be 45%, and the chitin obtained from fermented SHP (fSHP) displayed a similar characteristic Fourier-transform infrared spectroscopy (FTIR) profile as that from SHP. In addition, SHP, fSHP, and chitins obtained from SHP and fSHP were investigated for their adsorptive capacity of nine types of dyes, and chitin obtained from fSHP displayed a good adsorption rate on Congo Red and Red No. 7, at 99% and 97%, respectively. In short, the results provide potential support for the utilization of SHP in the production of *P. mucilaginosus* TKU032 protease via the fermentation as well as the preparation of chitin from fSHP as an effective dye adsorbent.

## 1. Introduction

Chitin is a natural polymer constituted by *N*-acetylglucosamine (GlcNAc) and *N*-glucosamine (GlcN) units where the number of GlcNAc units must be higher than 50% [1]. It occurs abundantly in the exoskeleton of arthropods, the gladius of squid, and the cell walls of fungi [2,3]. Chitin and its derivatives have been used in many fields; for instance, environmental treatment, agriculture, medicine, and food [4,5,6,7,8]. Chitinous fishery by-products, such as shrimp heads, shrimp shells, crab shells, and squid pens are the major sources for chitin and chitosan preparations [7,8,9,10,11,12]. These types of materials contain a considerable amount of protein [13]. In the process of extracting chitin, protein is conventionally removed from the raw materials by using a strong alkali, which could potentially lead to toxic wastewater emissions [14]. To develop eco-friendly techniques, numerous studies have focused on using proteolytic microorganisms to carry out the deproteinization step in chitin extraction. Accordingly, the protein component in chitinous materials can be degraded by proteolytic enzymes secreted during microbial fermentation in order to ultimately cause deproteinization. Additionally, valuable products such as proteases [15], prebiotics [16], and antioxidants [17] could be obtained from the fermentation of chitinous fishery by-products depending on the fermenter.

Proteases are a group of hydrolytic enzymes that cleave to the peptide bond in the protein structure [18]. They are found in various living creatures, including plants, animals, and microbes. Proteases are one of the major groups of industrial enzymes because of their extensive applications (e.g., leather, waste treatment, detergent, peptide synthesis, diagnostics, silk degumming, therapeutics, food processing, etc.) [19]. Among the sources of proteases, the microbes have a few advantages, such as being simple to scale for fermentation, requiring less space, and having a short growth period. In addition, microbes can consume various types of raw materials as a source of nutritional carbon and nitrogen (C/N) for protease production [19,20]; hence, the use of inexpensive and suitable materials to make cultural media would potentially reduce the cost of enzyme production. Accordingly, numerous studies have been conducted concerning the utilization of agricultural and fishery by-products for the production of protease [19,20,21,22,23,24]. Among the potential candidates, shrimp heads containing a large amount of protein can be used as good fermentation material [19,23]. 

*Paenibacillus* is a group of rod-shaped bacteria originally from the genus *Bacillus* [25]. Many strains from *Paenibacillus* can produce various bioactive compounds. The most significant of these are alpha-glucosidase inhibitors [26,27], chitinases/chitosanases [28,29,30,31,32], exopolysaccharides [33,34], antioxidants [34], and anti-inflammatory medication [35]. Surprisingly, proteases from *Paenibacillus* strains are rarely reported, while a large number of *Bacillus* proteases have been well explored and are being applied on an industrial scale. In addition, several *Paenibacillus* strains can synthesize proteases by using the culture media made from chitinous fishery by-products [28,29,31,32]. Hence, the exploration of protease production by using *Paenibacillus* strains is interesting, especially when using the by-products as the fermentation material. *P. mucilaginosus* TKU032 was isolated in the previous study by using the medium containing squid pens [34]. Out of 16 tested strains (including *Paenibacillus*, *Bacillus*, *Serratia*, *Lactobacillus*, and *Rhizobium* strains), *P. mucilaginosus* TKU032 yielded the best protease productivity on a medium containing demineralized crab shells, indicating the potential of this strain in protease production [31].

Since dyes are commonly used in many industries, such as the food industry, the textile industry, the paper pulp industry, etc., they are one of the most considerable contributors to water pollution. In addition, dye-containing wastewater is known to be potentially toxic to the environment and difficult to remove [36,37,38]. Several methods are effective in dye removal, such as oxidation, adsorption, ultrafiltration, and coagulation, and due to its high performance, flexibility, and minimal energy requirements, adsorption is one of the most appropriate methods for dye effluent treatment [38]. Among the dye adsorbents, chitin is considered to be the appropriate material due to its high dye adsorption potency, non-toxicity, low cost, and plentiful supply [39,40]. In order to maintain the low cost of dye adsorbent, chitinous residue from the microbial fermentation could be an interesting source [41,42,43], which gave rise to the idea of reusing the chitinous source discharged from the protease synthesis from *P. mucilaginosus* TKU032 to produce chitin as a dye adsorbent.

In the current study, several types of chitinous fishery by-products were utilized to cultivate *P. mucilaginosus* TKU032 and achieve high protease productivity. Afterward, the protease was isolated, purified, and characterized. To enhance the value of the fermentation, the fermented shrimp head powder (fSHP) obtained from the culture medium was used to prepare chitin. Finally, the ability of the obtained chitin to adsorb dyes was investigated.

## 2. Materials and Methods 

### 2.1. Materials

*P. mucilaginosus* TKU032 was isolated and identified in an earlier study [34]. Shin-Ma Frozen Food Co. (I-Lan, Taiwan) provided the chitinous fishery by-products (crab shells, squid heads, and shrimp shells), and Fwu-Sow Industry (Taichung, Taiwan) provided shrimp heads. Crab shells and shrimp shells were demineralized according to the method stated in a previous study [18]. In short, crab shells and shrimp heads were treated for two days with 2 N HCl solution and then rinsed with tap water to remove the residual acid. Eventually, the remaining solids were dried at 60 °C for 24 h. Tyrosine, *β*-mercaptoethanol, phenylmethylsulfonyl fluoride (PMSF), and Congo Red were bought from Sigma-Aldrich Corp. (St. Louis, MO, USA). Red No. 6 (Barium Lake), Yellow No. 4 (Tartrazine), Red No. 40 (Allura Red AC), Red No. 7 (Calcium Lake), Green No. 3 (Fast Green FCF), Yellow No. 5 (Sunset Yellow FCF), Blue No. 1 (Brilliant Blue FCF), and Blue No. 2 (Indigotine) were procured from First Cosmetics Works Company (Taipei, Taiwan). All other reagents used were of the highest grade available.

### 2.2. Protease Activity Test

An enzyme solution of 50 µL was mixed with 50 µL of casein (1%, *w/v*), and the mixture was kept at 37 °C for 30 min. To eliminate the enzyme activity and precipitate the residual casein, 300 µL of trichloroacetic acid (TCA) solution (0.19 M) was added to the mixture. The control tube had a similar composition, but the TCA solution was added immediately into the mixture of enzymes and casein before the incubation time took place. The precipitate was later removed by centrifuging at 13,000 rpm for 10 min, and the supernatant was collected. The amount of tyrosine in the enzyme reaction was detected by Folin’s Phenol reagent. One protease unit is the number of enzymes needed to liberate 1 µmol of tyrosine in one minute. 

### 2.3. Screening the Optimal Chitinous Fishery By-Products for Protease Production

One gram of each fishery by-product, including shrimp head powder (SHP), shrimp shell powder (SSP), demineralized shrimp shell powder (deSSP), demineralized crab shell powder (deCSP), and squid pen powder (SPP) was added to 100 mL of basal solution, which contained 0.1% K_2_HPO_4_ (*w/v*) and 0.05% MgSO_4_ (*w/v*) to prepare the medium for *P. mucilaginosus* TKU032 fermentation. Before being used, each medium was prepared in a 250 mL glass flask and sterilized at 121 °C for 20 min. Then, the seed solution was added to every media in a 1/100 (*v/v*) ratio. The fermentation was maintained at 150 rpm and 37 °C. After each specified day of incubation time, 1.0 mL of the liquid medium was withdrawn to determine the protease activity.

### 2.4. Purification of the Protease

*P. mucilaginosus* TKU032 was incubated as per the optimal conditions for protease production. The liquid supernatant, which was used for isolating the *P. mucilaginosus* TKU032 protease, was collected by centrifuging the culture medium at 6000 rpm for 10 min. Ammonium sulfate (60% *w/v*) was added to the liquid supernatant, and the mixture was kept at 4 °C for 24 h to precipitate the crude enzyme. Then, the crude enzyme was collected after centrifugation (9000 rpm, 30 min) and dissolved in 20 mM sodium phosphate buffer (pH 6). The residual ammonium sulfate in the crude enzyme solution was removed by using a cellulose dialysis membrane (20 mM sodium phosphate buffer for 24 h). Next, the enzyme solution was applied to the Macro-Prep High S column equilibrated by 20 mM sodium phosphate buffer (pH 6), and the elution was conducted by using a sodium chloride gradient (0–1 M). The fractions exhibiting protease activity were collected and concentrated by the lyophilization method. Finally, an HPLC system using the KW-802.5 column was used to purify the obtained enzyme (flow rate: 0.6 mL/min, detector: A_280 nm_, column temperature: 25 °C).

### 2.5. Effect of pH and Temperature

The optimal pH of *P. mucilaginosus* TKU032 protease was determined in the same manner as the above-described protease activity test by adjusting the pH of the reaction solution. The pH adjustment buffer system included sodium carbonate (pH 9–11), sodium phosphate (pH 6–8), sodium acetate (pH 5), and glycine HCl (pH 2–4). *P mucilaginosus* TKU032 protease’s pH stability was determined based on its residual activity after incubating the enzyme at various pH points, from pH 2 to 11, for 1 h. To determine the optimal temperature of the *P. mucilaginosus* TKU032 protease, the enzyme and casein solution mixture was maintained at various temperatures (10–100 °C) for 30 min, and then the enzyme activity was determined following the above-mentioned protease activity test. The thermal stability of the *P. mucilaginosus* TKU032 protease was determined based on its residual activity after incubating the enzyme at different temperatures (10–100 °C) for 1 h. The residual activity of the *P. mucilaginosus* TKU032 protease was examined at 60 °C after 30 min.

### 2.6. Substrate Specificity

The substrate specificity of *the P. mucilaginosus* TKU032 protease was tested on casein, elastin, myoglobin, fibrinogen, hemoglobin, bovine, serum albumin, gelatin, and keratin by using the conditions for the protease activity test (as described above). The protease activity in casein was used as the control.

### 2.7. Effect of Metal Ions, Inhibitors, and Surfactants

Chemicals were prepared at 5 mM concentration, and SDS, triton X-100, Tween 20, and Tween 40 were prepared at 10%. Initially, *P. mucilaginosus* TKU032 protease solution was added to each of those chemicals in the same proportion for 30 min, and then the residual activity of the enzyme was determined by the protease activity test (as described above).

### 2.8. Chitin Extraction

The chitinous samples were treated with NaOH solution (0.6 M) for 4 h in a water bath (60 °C) to remove protein. Later on, the samples were treated with HCl solution (0.6 M) for 4 h at room temperature to remove mineral salts; then, they were washed well with tap water to remove the residual acid. Then, a solvent mixture of ethanol and acetone (1/1, *v/v*) was used to remove the color in the samples. Eventually, the obtained chitins were dried in an oven at 60 °C for 24 h.

### 2.9. FTIR Analysis

The Fourier-transform infrared spectroscopy (FTIR) spectra of chitin samples were measured under the support of KBr. Initially, the sample and KBr were mixed and crushed carefully in an agate mortar. Later on, the mixture was pressed to form a homogeneous pellet. Finally, FTIR spectra were measured at a frequency range of 4000–400 cm^−1^ on an FTIR spectrophotometer (Nicolet *i*S5, Thermo, Waltham, MA, USA). 

### 2.10. Dye Adsorption Test

In a plastic falcon centrifuge tube, 20 mg of adsorbent was applied to 5 mL of dye solution (0.001%, *w/v*). The tube was later mounted on a rotary shaker and held for 60 min. Then, the residual dye in the mixture was measured through a spectrophotometer (the absorption wavelengths of Red No. 6, Yellow No. 4, Red No. 40, Red No. 7, Green No. 3, Yellow No. 5, Blue No. 1, and Blue No. 2 were 509, 425, 509, 509, 625, 480, 629, and 610 nm, respectively). The adsorption rate (%) was calculated by using the formula:

Adsorption rate (%) = (C-S)/C(1)

where C is the absorbance of the original dye solution, and S is the absorbance of the experimental group. 

## 3. Results and Discussion

### 3.1. Screening the Chitinous Fishery By-Products for Protease Production

**Figure 1:**
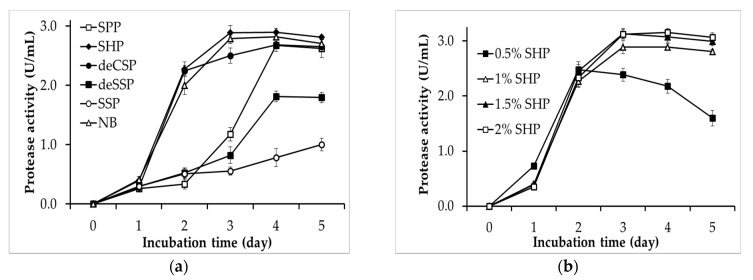
Effects of (**a**) temperature and (**b**) pH on the activity and stability of *P. mucilaginosus* TKU032 chitosanase. The error bar is the standard deviation of three repetitions.

Finding a suitable nutrient source for the growth and synthesis of the bioactivity products of microbial strains is an essential step [32]. Hence, the protease productivity of *P. mucilaginosus* TKU032 on medium containing different chitinous fishery by-products, including SHP, deCSP, deSSP, SSP, and SPP, was investigated herein. As shown in Figure 1, *P. mucilaginosus* TKU032 can synthesize protease on all tested culture mediums. Among the fishery by-products, SHP, SPP, and deCSP appeared to be highly suitable for the production of protease, and the *P. mucilaginosus* TKU032 protease productivities on those mediums were 2.89 ± 0.12 U/mL (day 3), 2.67 ± 0.1 U/mL (day 4), and 2.69 ± 0.12 U/mL (day 4), respectively. These results were higher than the protease productivity of *P. mucilaginosus* TKU032 on deSSP and SSP, with the maximum activity at 1.81 ± 0.09 U/mL (day 4) and 1.03 ± 0.1 U/mL (day 5), respectively. In addition, the protease productivity of *P. mucilaginosus* TKU032 on SHP could reach the maximum value after only three incubation days, whereas that using SPP and deCSP as substrates was achieved after four incubation days, indicating that SHP as the sole C/N source gives a better result of *P. mucilaginosus* TKU032 protease production than that by other chitinous fishery by-products. The protease productivity of *P. mucilaginosus* TKU032 was also examined on nutrient broth (NB), which is a commercial medium. It is clear that the maximum protease activities of the culture supernatants of SHP 2.89 ± 0.12 U/mL (day 3) and NB 2.79 ± 0.08 U/mL (day 3) were nearly similar, indicating that medium containing SHP could be an effective alternative. SHP is the waste from the fishery process, and it can be a cost-effective source. Indeed, several studies have used SHP as the nutrition source for producing various biological compounds, including proteases [16,18,33]. Hence, SHP was finally selected as the appropriate nutritional source for fermentation by *P. mucilaginosus* TKU032 to produce proteases.

The effect of SHP concentration on the protease productivity of *P. mucilaginosus* TKU032 is presented in Figure 1b. The maximum protease activity of the culture supernatants with 0.5%, 1%, 1.5%, and 2% SHP were 2.48 ± 0.1 U/mL (day 2), 2.89 ± 0.12 U/mL (day 3), 3.14 ± 0.1 U/mL (day 3), and 3.15 ± 0.07 U/mL (day 4), respectively, indicating that a higher concentration of SHP is more appropriate for higher *P. mucilaginosus* TKU032 protease productivity. Furthermore, there was no significant difference in the maximal protease activity of 1.5% SHP (3.14 U/mL) and 2% SHP (3.15 U/mL), suggesting that 1.5% SHP could produce the highest amount of the protease and cost less to prepare the medium rather than 2% SHP. Finally, 1.5% (*w/v*) was selected as the optimum SHP concentration for protease production by *P. mucilaginosus* TKU032.

### 3.2. Purification of the Protease

**Figure 2:**
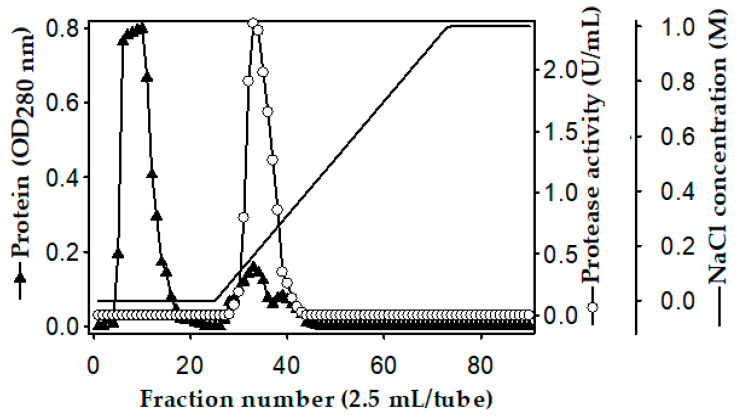
Ion-exchange chromatography profile of the *P. mucilaginosus* TKU032 crude enzyme on the Macro prep High S column.

The purification of the *P. mucilaginosus* TKU032 protease was carried out on the 3-day culture medium. The ion-exchange profile on the Macro-Prep High S column of the *P. mucilaginosus* TKU032 crude enzyme is shown in Figure 2. Most of the protein in the crude enzyme solution was washed out at the washing stage by using a sodium phosphate buffer (pH 6, 20 mM), and only one proteolytic activity was observed at the elution stage, indicating that the protease had a p*I* higher than pH 6, and thus, could bind to the resin. The fractions showing activity (tube numbers 29 to 37) were pooled, dialyzed against the buffer, and lyophilized before applying the next purification step. The purification process was carried out by the gel filtration method using a high-performance liquid chromatography (HPLC) system with a KW-802 column. The recovery yield of the obtained protease was 15.22% with 33.69 folds of the specific activity (Table 1).

**Figure 3:**
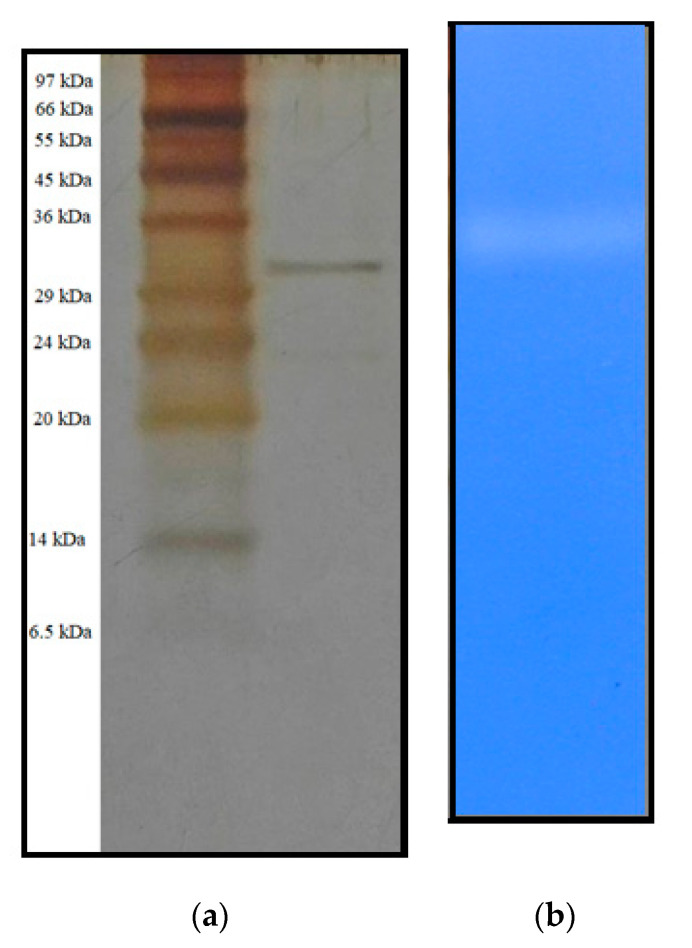
SDS-PAGE (**a**) and zymography (**b**) profiles of purified TKU032 protease.

The homogeneity of the purified protease was determined through SDS-PAGE and silver staining analysis. As shown in Figure 3a, after the HPLC step, a single protease protein band of molecular weight (MW) 32 kDa (approximately) was observed at the lane of pool fraction. This is consistent with other reports, which showed the MW around 32 to 60 kDa (Table 2). The activity of the obtained protease was detected by zymogram analysis, and a proteolytic band was observed on the gel containing 0.2% gelatin (Figure 3b). Therefore, these observations could confirm that the *P. mucilaginosus* TKU032 protease was successfully purified. 

### 3.3. Effect of pH and Temperature on Enzyme Activity and Stability

**Figure 4:**
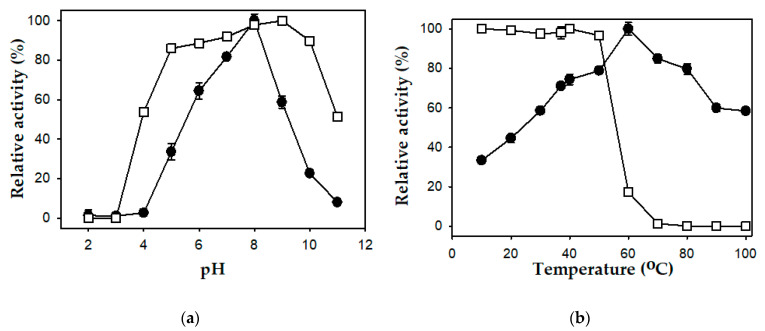
Effects of pH (**a**) and temperature (**b**) on the activity (●) and stability (□) of the *P. mucilaginosus* TKU032 protease. The error bar is the standard deviation of three repetitions.

The effect of pH on the activity and stability of the *P. mucilaginosus* TKU032 protease was tested in a pH range of 2–11. The enzyme was maximally active in the pH range of 5 to 10 and mostly inactive at pH 2, 3, 4, and 11 (Figure 4a). A linear increase in activity was observed from pH 4 to 8 and significantly declined at higher pH. This result indicated that the *P. mucilaginosus* TKU032 protease was optimally active at pH 8, which was lower than that of alkaline proteases from *Paenibacillus* sp. TKU047 and *P. tezpurensis* sp. nov. AS-S24-II [18,44]. The stability of the protease was maximum in the range from pH 5 to 10. 

The activity of *P. mucilaginosus* TKU032 was determined over a range of temperatures (10–100 °C) and a linear increase in activity was observed from 10 to 60 °C. At 60 °C, the activity of the protease was the highest, indicating that this was the optimum temperature for the enzyme. There was a significant decline in enzyme activity at higher temperatures (84.88% at 70 °C, 79.70 at 80 °C, 59.87 at 90 °C, and 58.37 at 100 °C) when compared with the enzyme activity at 60 °C (Figure 4b). This result was comparable with that of some commercial proteases, such as Maxatase, Savinase, and Alcalase, which have the optimum temperature at 50–60 °C [54]. The *P. mucilaginosus* TKU032 protease possessed a higher optimum temperature than the proteases from *P. lautus* [47] and *P. tezpurensis* sp. nov. AS-S24-II [44], but it was lower than that of *Paenibacillus* sp. TKU047 [18]. The thermal stability test was performed by incubating the enzyme solutions at different temperatures for 60 min. Figure 4b showed that the *P. mucilaginosus* TKU032 protease was stable up to 50 °C, with a gradual decline in enzyme activity at higher temperatures. Particularly, the enzyme retained only 17.30% of the initial activity at 60 °C and completely lost its activity at higher temperatures. 

### 3.4. Substrate Specificity

The activity of the *P. mucilaginosus* TKU032 protease on different protein substrates is presented in Table 3. The activity was in the order of casein > bovine serum albumin > fibrinogen > gelatin > myoglobin > keratin. The enzyme showed no activity on elastin and hemoglobin. The results indicate that the *P. mucilaginosus* TKU032 protease exhibits good caseinolytic activity. The highest activity on casein was exhibited by the *Paenibacillus* sp. TKU047 protease [18], while the highest keratinase activity was exhibited by the *P. tezpurensis* sp. nov. AS-S24-II protease [44].

### 3.5. Effect of Metal Ions, Inhibitors, and Surfactants

The effect of metal ions, enzyme inhibitors, and surfactants on the activity of the *P. mucilaginosus* TKU032 protease is examined in Table 4. Metal ions such as Zn^2+^, Mg^2+^, Ca^2+^, and Fe^2+^ did not show a significant effect on the enzyme activity. Ba^2+^ was slightly inhibited, while Cu^2+^ strongly inhibited the *P. mucilaginosus* TKU032 protease with residual activities of 95.69% and 19.68%. Among the tested metal ions, only Na^+^ caused a slight increase in the activity of the *P. mucilaginosus* TKU032 protease (108.5%). The enzyme showed high resistance to non-ionic surfactants (Triton X-100, Tween 20, and Tween 40) and retained most of its activity at 86.77%, 100.75%, and 107.37%, respectively. However, a strong ionic surfactant, such as Sodium Dodecyl Sulfate (SDS) could dramatically decrease the enzyme activity to only 22.07% of its normal activity. The reducing agent (*β*-mercaptoethanol) partially affected the enzyme activity by decreasing it to 72.95%. Ethylenediaminetetraacetic acid (EDTA), a metalloprotease inhibitor, exhibited the highest inhibitory effect on the activity of the *P. mucilaginosus* TKU032 protease, and the enzyme retained only 8.21% of its activity. Meanwhile, phenylmethylsulfonyl fluoride (PMSF), a serine protease inhibitor, showed a moderate effect on the enzyme activity, with a residual activity of 75.08%. This indicates that *P. mucilaginosus* TKU032 is a metalloprotease. Several metalloproteases from the *Paenibacillus* genus have been reported [18,48].

### 3.6. Chitin Recovery from fSHP

In the chitin extraction process, fermentation using protease-producing bacterial strains is one of the common biological methods for deproteinization [11]. In this study, the fermentation using *P. mucilaginosus* TKU032 could achieve high protease productivity and partially remove the proteins in SHP. Indeed, the ratio of proteins in fSHP was found to be significantly lower than that in SHP (28.45 ± 1.3%, and 52.11 ± 0.85%, respectively), suggesting that the deproteinization rate of the fermentation could be calculated at 45.44 ± 2.5%. While the deproteinization rate of fishery chitinous materials used for the microbial fermentation varied, in general, the proteins could not be removed completely. Consequently, to produce high-quality chitin, the residual proteins and minerals need to be removed in further steps [11]. In this study, HCl (0.6 M) and NaOH (0.6 M) were used to conduct the demineralization and deproteinization steps. Eventually, a solvent mixture of ethanol and acetone (1/1, *v/v*) was used to remove the color of the obtained chitin. The chitin recover yield from fSHP was calculated at 20.67 ± 0.47% and achieved a recovery rate (compared to the ratio of chitin in SHP) of 89.24 ± 2.03%. In general, the chitin recovery yield from shrimp wastes occurs in a range of 5–23% [7], which means that the yield from fSHP is acceptable. 

**Figure 5:**
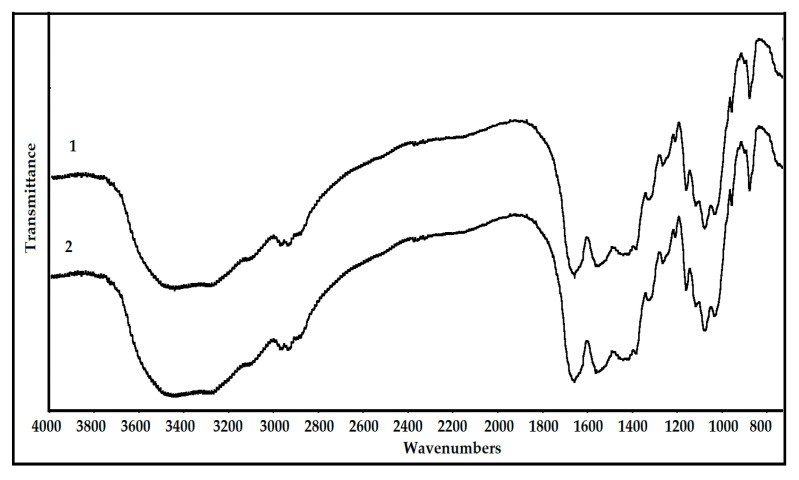
Fourier-transform infrared spectroscopy (FTIR) profile of chitin from shrimp head powder (SHP) (1), and chitin from fSHP (2).

Fourier-transform infrared spectroscopy (FTIR) analysis was used to confirm the quality of the chitin product. Figure 5 shows that chitins obtained from SHP and fSHP media were identified as *α*-chitin with the indicator peaks at 3450 cm^−1^ (ν(OH) association in the pyranose ring), 3278 cm^−1^ (ν(NH) association in *trans*-secondary amides), 3110 cm^−1^ (ν(NH) association in *cis*- and *trans*-secondary amides), 2960 cm^−1^ (ν_as_(CH_3_) in the NHCOCH_3_ group), 2930 cm^−1^ (ν_as_(CH_2_) in the CH_2_OH group), 2880 cm^−1^ (ν(C-H) in the pyranose ring), 1621–1661 cm^−1^ (ν(C=O) in the NHCOCH_3_ group), 1550 cm^−1^ (ν(C–N) + δ(N-H) in the NHCOCH_3_ group), 1420 cm^−1^ (δ(CH_2_) in the CH_2_OH group), 1380 cm^−1^ (δ_s_(CH_3_) in the NHCOCH_3_ group), 1320 cm^−1^ (δ(C–H) in the pyranose ring), 1260 cm^−1^ and 1205 cm^−1^ (complex vibrations of the NHCO group), 1155 cm^−1^ (ν(C–N) + δ(N–H) in the NHCOCH_3_ group), 1118 cm^−1^ (ν_as_(C–O–C) glycosidic linkage), 1074 cm^−1^ (ν(C–O) in the secondary OH group), 1027 cm^−1^ (ν(C–O) in the primary OH group), 958 cm^−1^ (pyranose ring skeletal vibrations), and 877 cm^−1^ (ring streching β-1,4 glycosidic bonds) [55,56]. The FTIR results of chitins from SHP and fSHP did not show any significant difference, indicating that *P. mucilaginosus* TKU032 fermentation did not significantly alter the structure of chitin in SHP. As a result, chitin extraction from fSHP by *P. mucilaginosus* TKU032 fermentation to produce protease could be feasible. 

### 3.7. Dye Adsorption by Chitin from fSHP

**Figure 6:**
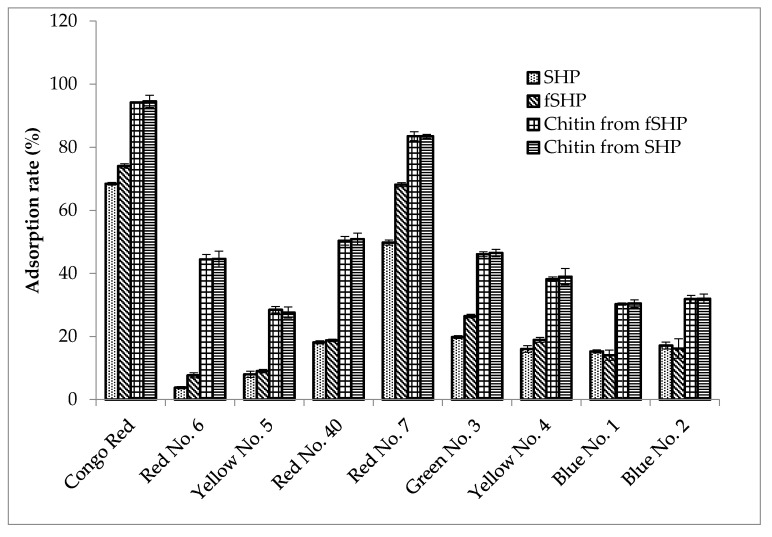
Dye adsorption ability of SHP, fermented SHP (fSHP), chitin from SHP, and chitin from fSHP. The error bar represents the standard deviation of three repetitions.

The textile and food industries consume a significant amount of dyes to color their products, and as a result, a great amount of dye-containing wastewater is discarded during their manufacturing processes [57,58]. Among various technologies used to remove dye from wastewater (including physicochemical, biological, and chemical methods), adsorption is one of the most efficient. By-products from agriculture and fishery processes are suitable adsorbents because of their low cost, abundance, and effectiveness. Moreover, some modifications can be easily conducted on these materials in order to achieve a higher adsorption capacity [41,42,43,59,60,61]. In the current study, the dye adsorption ability of chitins from fSHP and SHP was investigated and compared with that of SHP and fSHP. According to Figure 6, fSHP showed a higher rate of adsorption of dyes, including Congo Red, Red No. 6, Red No. 7, Green No. 3, and Yellow No. 5, as compared to SHP (74.06 ± 0.69% and 68.45 ± 0.43%; 7.66 ± 0.79% and 3.78 ± 0.36%; 68.15 ± 0.62% and 49.78 ± 0.83%; 26.46 ± 0.57% and 19.81 ± 0.67%; 18.91 ± 0.78% and 16.04 ± 1.07%, respectively). This indicates that the bacterial activity during the fermentation may alter the structure of the SHP, making it more effectively absorbable. Likewise, fermented SPPs by *B. cereus* strains could express better adsorption ability on Disperse Black 30, Disperse Blue 60, Disperse Yellow 54, Disperse Red 60, Tartrazine, and Allura Red AC [41]. Interestingly, chitins from both SHP and fSHP expressed nearly similar and high adsorption ability on all tested dyes (94.57 ± 1.95% and 94.21 ± 0.21% on Congo Red; 44.58 ± 2.51% and 44.44 ± 1.55% on Red No. 6; 27.58 ± 1.76% and 28.48 ± 1.01% on Yellow No. 5; 50.87 ± 1.91% and 50.33 ± 1.37% on Red No. 40; 83.48 ± 0.63% and 83.53 ± 1.4% on Red No. 7; 46.48 ± 1.16% and 46.07 ± 0.75% on Green No. 3; 38.95 ± 2.58% and 38.17 ± 0.72% on Yellow No. 4; 30.44 ± 1.2% and 30.29 ± 0.34% on Blue No. 1; and 31.97 ± 1.45% and 31.85 ± 1.17% on Blue No. 2, respectively). Therefore, chitin played an important role in adsorbing the tested dyes. The mechanism of dye adsorption by chitin may be related to ionic interaction between the functional groups (such as amino and hydroxyl groups) on the chitin structure and anion groups on dyes (such as R–SO_3_^−^) [41,42,43]. In addition, there was no significant difference between the adsorption rates of chitin from fSHP and SHP, possibly indicating the structural similarity of chitinous materials obtained from SHP and fSHP. 

**Figure 7:**
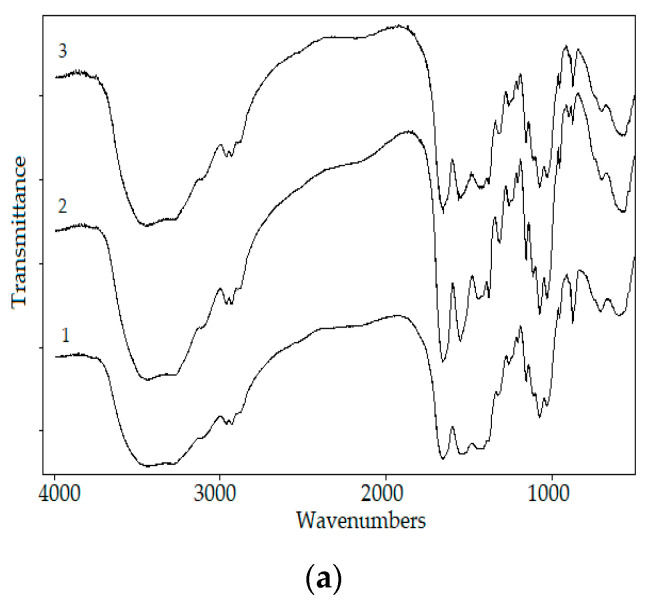

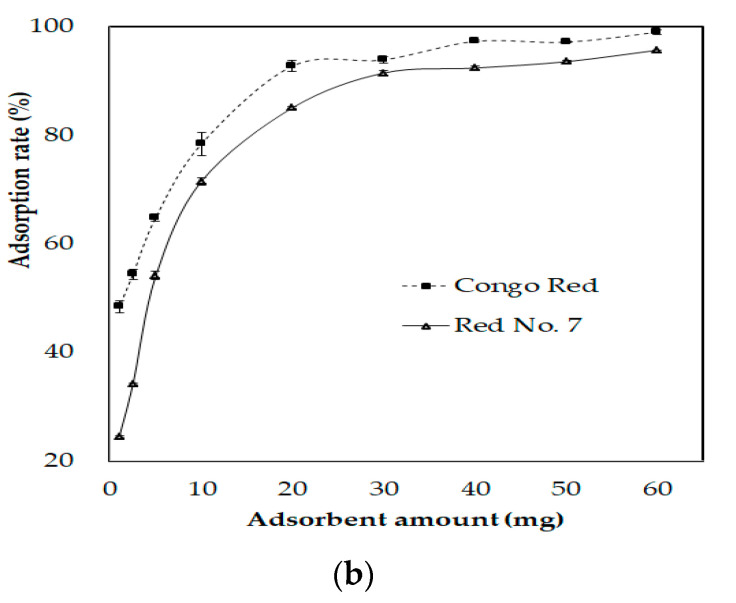
Effect of the chitin amount on the adsorption of Congo Red and Red No. 7 (**a**), and Fourier-transform infrared spectroscopy (FTIR) profiles of chitin before and after adsorbing the dyes (**b**). 1, chitin; 2, chitin after adsorbing Congo Red; 3, chitin after adsorbing Red No. 7. The error bar represents the standard deviation of three repetitions.

The chitin from fSHP showed more than an 80% adsorption rate of Congo Red and Red No. 7; therefore, this material holds great potential in removing those two dyes from wastewater. According to Figure 7, 1.39 and 5.14 mg of chitin could remove 50% of Congo Red and Red. No. 7, respectively. The maximal Congo Red and Red No. 7 adsorption rates of chitin were calculated to be 99.06 ± 0.97% and 97.26 ± 0.78%, respectively. The FTIR profiles of chitin from fSHP before and after the absorption of Congo Red and Red No. 7 revealed some change in their absorption intensity. The intensity increase where the peaks are at 1658 cm^−1^ could be attributed to –N=N– stretching, 1550 cm^−1^ could be due to amino groups from Congo Red, 1380 cm^−1^ could be due to the presence of N–H, which may be formed by hydrogen bonding between chitin hydroxyl groups and the azoic group on dyes [60], and the peak at 1150 cm^−1^ could be due to –SO_3_ groups. Some other changes in the absorption intensity of chitin before and after the adsorption process could also be observed at 1320, 2934, 2963, 950, 901, and 871 cm^−1^. Hence, the FTIR results confirmed the adsorption of Congo Red and Red No. 7 by the chitins.

Shrimp head waste is a generally cheap, raw material that is abundantly discharged from the seafood processing industry. Therefore, the products produced from the microbial conversion of that material could potentially gain a cheaper price. As a result of the great interest, SHP has been used to produce various bioactive compounds, such as protease [5,18,23], chitosanase [62], α-glucosidase inhibitor [63], and nattokinase [64]. Indeed, in this research, SHP is a potential C/N source for protease production by *P. mucilaginosus* TKU032, and fSHP can also be recovered from the culture broth for environmental applications such as the removal of organic dyes. The good adsorptive capacity of chitin from fSHP with dyes may have potential applications in the field of water treatment. 

## 4. Conclusions

Research on the conversion of chitinous fishery waste to high-quality products is ongoing. In this study, the effective production of protease by *P. mucilaginosus* TKU032 was successfully established on a simple, low-cost medium by using shrimp heads as the sole source of C/N. Accordingly, a 32 kDa protease was isolated and purified by using the SHP culture medium. A significant amount of protein in SHP could be removed through *P. mucilaginosus* TKU032 fermentation, indicating that the extraction of chitin could be deployed to recover chitin from fSHP. In addition, chitins obtained from fSHP and SHP showed no significant difference in quality, indicating a noticeable utilization of residual SHP in the culture medium for chitin production. Additionally, the chitinous materials (fSHP and SHP chitins) exhibited good dye absorbing ability, especially on Congo Red and Red No. 7, making them potentially useful in water treatment plans.

## Figures and Tables

**Table 1:** Purification characteristics of the protease from *P. mucilaginosus* TKU032.

Step	Total Protein (mg)	Total Activity (U)	Specific Activity (U/mg)	Recovery (%)	Purification (fold)
**Cultural supernatant**	1237.25	1079.01	0.87	100.00	1.00
**(NH_4_)_2_SO_4_ precipitation**	125.42	867.25	6.91	80.38	7.93
**Macro-Prep High S**	29.63	251.20	8.48	23.28	9.72
**KW-802.5**	5.59	164.27	29.38	15.22	33.69

**Table 2:** Characteristics of protease from the *Paenibacillus* genus.

Enzyme/Strain	MW	Optimum		Stability		Best C/N Source	Ref.
		pH	Temp.	pH	Temp.		
Protease *P. mucilaginosus* TKU032	32	8	60	5–10	≤50 °C	SHP	This study
Alkaline protease *P. tezpurensis* sp. nov. AS-S24-II	43	9.5	45–50 °C			Casein	[44]
Alkaline protease *Paenibacillus* sp. TKU047	32	9	70–80 °C	6–11	≤60 °C	SHP	[18]
Protease *Paenibacillus* sp. TKU042	35					SPP	[31]
Keratinase *P. woosongensis* TKB2						Feather and rice straw	[45]
Protease *Paenibacillus* spp. BD3526	35					Wheat bran	[46]
CtpA *P. lautus*	51.94	8–9	30 °C	8–9	≤35 °C	Skim milk	[47]
Metalloprotease *P. larvae*	59						[48]
Extracellular proteolytic enzymes *P. peoriae* NRRL BD-62 *						Thiamine, biotin, and nitrogen (TBN) broth	[49]
Extracellular proteolytic enzymes *P. polymyxa* SCE2 *						TBN broth	[49]
Pro-Pro endopeptidase *P. alvei*							[50]
Fibrinolytic enzyme *Paenibacillus* sp. IND8						Wheat bran, yeast extract, and sucrose	[51]
Protease *P. riograndensis* SBR5							[52]
Protease *P. polymyxa* E681							[53]

* Four proteolytic bands were detected on gelatin–SDS-PAGE (20, 35, 50, and 210 kDa).

**Table 3:** Substrate specificity of the *P. mucilaginosus* TKU032 protease.

	Relative Activity (%)
Casein	100.00 ± 15.42
Elastin	0
Myoglobin	35.14 ± 12.71
Fibrinogen	48.65 ± 8.75
Hemoglobin	0
Bovine serum albumin	62.16 ± 11.13
Gelatin	40.54 ± 8.75
Keratin	20.08 ± 5.35

The data is presented as mean ± standard deviation.

**Table 4:** Effect of various chemicals on the activity of the TKU032 protease.

Chemical	Relative Activity (%)
None	100.00 ± 2.57
Cu^2+^	19.68 ± 1.89
Zn^2+^	98.16 ± 1.88
Mg^2+^	98.49 ± 1.90
Na^+^	108.50 ± 4.64
Ba^2+^	95.69 ± 1.07
Ca^2+^	100.21 ± 1.17
Fe^2+^	100.21 ± 2.89
EDTA	8.21 ± 4.06
PMSF	75.08 ± 4.33
*Β*-mercaptoethanol	72.95 ± 4.22
SDS	22.07 ± 3.27
Triton X-100	86.77 ± 4.84
Tween 20	100.75 ± 2.02
Tween 40	107.37 ± 2.46

EDTA, Ethylenediaminetetraacetic acid; PMSF, Phenylmethylsulfonyl fluoride; and SDS, Sodium dodecyl sulfate. The data are presented as mean ± standard deviation.
